# Perceived Communication and Cooperation with Physicians and Nurses and Occupational Outcomes Among Medical Social Workers in China: A Cross-Sectional Study

**DOI:** 10.3390/healthcare14131839

**Published:** 2026-06-24

**Authors:** Congde Xu, Jinlin Pang, Zhen Li

**Affiliations:** Department of Social Work, School of Law, Qingdao University of Science and Technology, Qingdao 266061, China

**Keywords:** medical social work, communication and cooperation, physicians, nurses, occupational outcomes, China, cross-sectional study

## Abstract

Background/Objectives: Medical social work is performed in hospital teams, but evidence remains limited on how medical social workers’ perceived communication and cooperation with physicians and nurses are associated with occupational outcomes. Methods: Using the medical social work module of the China Social Work Longitudinal Survey 2019 (CSWLS2019), this cross-sectional study examined job satisfaction, personal accomplishment, self-rated service quality, and emotional exhaustion. We constructed a four-item communication-and-cooperation index and estimated ordinary least squares (OLS) models with HC3 heteroskedasticity-robust standard errors and city fixed effects. Robustness and exploratory supplementary checks assessed sample definition, alternative specifications, single-item ordered logit models, decomposed components, moderation, and a supplementary seemingly unrelated regression (SUR) system. Results: The index was positively associated with job satisfaction (*b* = 0.260, *p* = 0.0010), personal accomplishment (*b* = 0.416, *p* = 0.0335), and self-rated service quality (*b* = 0.151, *p* = 0.0275). Its association with emotional exhaustion was negative but not statistically significant in the main model (*b* = −0.186, *p* = 0.1207), although it became significant in the stricter sample. Decomposed and moderation models provided limited evidence for stable component-specific or moderation patterns. Conclusions: The findings should be interpreted as exploratory associational evidence rather than causal effects. Perceived communication and cooperation with physicians and nurses appear more consistently linked to favorable occupational evaluations than to emotional exhaustion among medical social workers in China.

## 1. Introduction

Medical social work in China is increasingly embedded in hospital-based and integrated-care teams, where psychosocial assessment, case management, crisis intervention, resource linkage, and discharge planning require routine coordination with physicians, nurses, and other professionals [1,2,3,4,5,6,7,8,9]. This setting gives medical social workers a distinctive position: they connect clinical processes with patients’ social circumstances, yet their role is often negotiated inside organizations dominated by medical and nursing logics [10,11,12,13]. Evidence from hospital and integrated-care settings shows that social workers’ contributions depend on whether colleagues understand their role, communicate with them, and cooperate in moving cases forward [9,10,11,12,13]. Its development has been shaped by uneven local institutionalization, including documented experience from Shanghai and recent accounts of integrated development of medical social work in China [1,2].

In this study, we use the term perceived communication and cooperation with physicians and nurses to describe the empirical indicator. This is narrower than the full multidimensional construct of interprofessional collaboration, which may also include role clarity, mutual respect, shared goals, team acceptance, and organizational support [14,15,16,17,18]. The four survey items used here capture the interactional team interface reported by medical social workers: communication with physicians, physician cooperation, communication with nurses, and nurse cooperation. Broader collaboration concepts are therefore used as contextual and interpretive background, not as directly measured dimensions.

Against this background, the study asks two research questions: RQ1: How is medical social workers’ perceived communication and cooperation with physicians and nurses associated with job satisfaction, personal accomplishment, self-rated service quality, and emotional exhaustion? RQ2: Are these associations more consistent for favorable occupational evaluations than for emotional exhaustion? The contribution is empirical and comparative. The study does not propose a new theory, dataset, measurement instrument, or causal design; rather, it compares four occupational outcomes side by side within the same cross-sectional analytical framework. This positioning also distinguishes the study from recent Chinese medical social work research focused primarily on burnout pathways [19].

The next section reviews the literature and sets out the analytical framework. We then describe the data and models, present the results, and discuss what the findings do and do not support.

## 2. Literature Review and Analytical Perspective

### 2.1. Medical Social Work and Role Positioning in Interprofessional Teams

Medical social work is embedded in organizational practice. Prior studies show that social workers in hospital-based, integrated, and team-based care assume responsibilities in care coordination, psychosocial assessment, resource linkage, and interventions addressing social determinants of health [3,4,6,20]. They are not peripheral to service delivery; they work at the intersection of clinical processes and patients’ social circumstances.

This position is not always secure. Research repeatedly notes that role boundaries, professional visibility, and team acceptance vary by organizational context and that social workers in interprofessional teams may encounter role ambiguity, power asymmetries, and communication barriers [10,11,12]. For medical social workers, the collaborative team interface is therefore both a condition for practice and a setting in which occupational strain may develop.

### 2.2. Conceptualizing Communication and Cooperation with Physicians and Nurses

Research on interprofessional collaboration has produced a substantial body of health care literature. Systematic reviews describe collaboration as a multidimensional arrangement involving communication, role clarity, shared goals, mutual respect, and team negotiation [14,15,16]. For medical social workers, these dimensions matter because their work often depends on whether physicians and nurses recognize social work knowledge, include social workers in team communication, and cooperate around patients’ social and discharge-related needs [10,11,12,13,21,22,23].

The present measurement is deliberately narrower than this broader conceptual field. The four survey items do not measure the full structure of interprofessional collaboration; they measure perceived communication and cooperation with physicians and nurses. We therefore avoid treating the index as a complete collaboration scale. In the analysis and discussion, broader concepts such as role clarity, team acceptance, and professional identity are used to interpret why communication and cooperation may matter, while the empirical claims remain tied to the four-item indicator.

### 2.3. Occupational Outcomes: Positive Evaluations and Occupational Strain

Research on the health and social care workforce commonly distinguishes between positive occupational evaluations and occupational strain. The first group includes job satisfaction, sense of accomplishment, and self-rated quality-related perceptions. The second includes psychological depletion, especially emotional exhaustion and burnout. Teamwork is often associated with higher job satisfaction and more favorable self-rated quality evaluations [24,25,26], and patient-centered work has linked interprofessional collaboration to self-rated quality-related outcomes [27,28]. Emotional exhaustion, by contrast, is usually treated as the product of several forces, including workload, role conflict, organizational resources, and structural pressure [29]. Social work studies likewise connect job satisfaction, burnout, and turnover-related outcomes to role stress, workload, supervisory conditions, and organizational environment rather than to a single source of strain [30,31,32].

We examine these four outcomes together because they represent two related but analytically distinct sides of occupational experience. Job satisfaction, personal accomplishment, and self-rated service quality are evaluative appraisals of work: whether workers feel satisfied, effective, and able to deliver good service. Emotional exhaustion reflects strain and depletion in the same practice environment. Placing the outcomes in one design allows us to ask whether communication and cooperation with physicians and nurses is a general correlate of occupational well-being or is more closely tied to role enactment and favorable work evaluation than to strain reduction.

This distinction guides the analysis. Communication-and-cooperation experience may be more consistently associated with job satisfaction, personal accomplishment, and self-rated service quality than with emotional exhaustion, which may depend more heavily on the broader organizational and task environment.

### 2.4. Interpreting Collaboration Through a Work-Resource Perspective

We use a work-resource perspective to structure the analysis. From this perspective, communication-and-cooperation experience is a team-level resource that may provide information, operational support, role recognition, and contextual backing. Organizational support, professional identity, and autonomy or organizational fit may also function as work resources because they are related to occupational outcomes and shape how the physician/nurse interface is experienced and managed [33,34,35]. This evidence is drawn from adjacent health care work-resource literature as well as social work research. Studies of hospital work environments link organizational context to job satisfaction, quality perceptions, and burnout-related outcomes [36]. Research on social work retention and integrated-care coordination similarly connects low social support, weak organizational and professional commitment, and high stress to withdrawal-related outcomes, whereas supervisory and team support appear protective for job satisfaction and burnout risk [37,38].

We use this perspective as an interpretive lens, not as a tested causal mechanism. The data are cross-sectional, and the medical subsample is based on convenience sampling, so temporal ordering among communication-and-cooperation experience, work resources, and occupational outcomes cannot be established. The aim is narrower: to examine whether communication-and-cooperation experience is systematically associated with different occupational outcomes and whether the associations vary across outcome dimensions. In this respect, the study complements work organized around burnout pathways or moderated mediation models. Rather than explaining burnout alone, it examines heterogeneity in the associations between communication-and-cooperation experience and multiple occupational outcomes and considers what those differences imply for medical social work practice.

## 3. Data, Sample, and Variables

### 3.1. Data Source

We use data from the Civil Affairs and Medical Social Worker Questionnaire of the China Social Work Longitudinal Survey 2019 (CSWLS2019). CSWLS2019 is a survey of the Chinese social work workforce, and the civil affairs/medical social work questionnaire includes a medical social work module. According to the survey documentation available to the authors, this portion of the sample was collected primarily through convenience sampling across multiple local survey sites; it should not be interpreted as a nationally representative sample of all medical social workers in China. The analysis is therefore designed to examine associational patterns within the module-completing medical social work sample rather than to estimate population prevalence.

### 3.2. Sample Definition

Our main analytical sample consists of respondents who answered the core medical social work items Q2–Q5, yielding 262 cases. We use this definition because Q2–Q5 directly measure respondents’ reported communication and cooperation with physicians and nurses, and identify respondents who completed the medical social work collaboration module. This design may overrepresent social workers who were more institutionally connected, more professionally active, or more exposed to physician/nurse interaction than social workers outside the module. To assess sensitivity to sample definition, we also construct a stricter sample that requires respondents in the main sample to satisfy at least one medical-condition indicator from the survey routing variable or the service-field marker. This stricter sample includes 249 cases.

A pre-estimation diagnostic check shows effective sample sizes of 237, 237, 237, and 236 across the four main outcome models. Sample loss in the main models is driven primarily by missing income, with smaller losses from education, role indicators, years of practice, and outcome-specific missingness. We use model-specific complete-case analysis: each model retains cases with non-missing values on the outcome, the communication-and-cooperation index, the relevant covariates, and city indicators. Appendix A Table A1 compares sample construction and missing-data decisions, including income and support-related missingness. In response to the reviewers’ concern about complete-case bias, we additionally estimated inverse-probability weighted (IPW) sensitivity models for complete-case inclusion. The IPW models use stabilized weights from outcome-specific ridge-logistic selection models based on variables observed in the full main sample, including the communication-and-cooperation index, gender, age, medical-module response indicators, number of valid collaboration items, and city indicators. Multiple imputation was considered but not used as the primary sensitivity approach because missingness is limited in the main outcomes but partly tied to module routing, income validity, and sparse city cells in a small medical-module sample. Complete-case and IPW estimates may still be biased if missingness depends on unobserved employment conditions or organizational setting; this is treated as a limitation. Accordingly, the IPW analysis is used only as a sensitivity check for observed complete-case selection, not as evidence that missingness was random or fully ignorable.

### 3.3. Outcome Variables

The study considers four occupational outcomes. Job satisfaction is measured with the core five-item scale (job_sat_core), which averages items L1A, L1B, L1C, L1E, and L1G, with higher values indicating greater job satisfaction. Emotional exhaustion is measured as the row mean of N1A–N1I (emo_exhaust), where higher values indicate greater exhaustion. Personal accomplishment is measured as the row mean of N3A–N3H (personal_accomp), where higher values indicate stronger perceived professional value and work effectiveness. Self-rated service quality is measured as the row mean of L2A–L2I (service_quality), where higher values indicate a more favorable evaluation of one’s own self-rated service quality. These scales are survey-based measures rather than newly validated instruments for this study. The emotional exhaustion and personal accomplishment items resemble burnout dimensions, but we treat them as survey-specific approximations rather than a full Maslach Burnout Inventory scale. Internal consistency was high for complete item responses in the main sample: communication-and-cooperation index (Cronbach’s *α* = 0.882), job satisfaction (*α* = 0.877), emotional exhaustion (*α* = 0.931), personal accomplishment (*α* = 0.954), and self-rated service quality (*α* = 0.891).

### 3.4. Key Independent Variable

The key explanatory variable is a four-item perceived communication-and-cooperation index (collab_index). It is constructed from smooth communication with physicians (Q2), physicians’ degree of cooperation (Q3), smooth communication with nurses (Q4), and nurses’ degree of cooperation (Q5). The original items are reverse-coded so that higher values indicate more positive communication and cooperation experiences, and the four items are then averaged. To examine whether any single component drives the overall pattern, supplementary decomposed models retain communication with physicians (doc_comm), cooperation from physicians (doc_coop), communication with nurses (nurse_comm), and cooperation from nurses (nurse_coop). These component models are treated as exploratory checks and do not serve as the basis for the main conclusions.

### 3.5. Supplementary and Control Variables

Supplementary analyses include several organizational and occupational perception variables to examine the boundaries of the focal association. Work support (support_index) is constructed from items in module I; values recorded as 0.0 in the questionnaire are treated as invalid and recoded to missing. Autonomy and organizational fit (autonomy_fit) are constructed from module G1, and professional identity (prof_identity) from module F2. These variables are used as supplementary moderators or parallel explanatory factors, not as identified causal mechanisms.

The control set includes gender, age, education, frontline service role, management role, supervisory role, years of social work practice, monthly income, contract type, labor dispatch status, formal establishment status, and city fixed effects. In the main tables, monthly income is rescaled per 1000 yuan to make the coefficients interpretable; log(income + 1) is used as an alternative income specification in robustness checks. Because the public dataset does not provide complete information on hospital type, hospital level, department, caseload, administrative burden, supervision quality, or organizational resources, these factors cannot be directly controlled. Contract type, dispatch status, formal establishment status, and city fixed effects are used as partial institutional proxies, but omitted institutional and workload variables remain important confounders.

### 3.6. Analytical Strategy

The main models are estimated with ordinary least squares (OLS), HC3 heteroskedasticity-robust standard errors, and city fixed effects. OLS is used because the outcomes are averaged multi-item Likert-type scales and are treated as approximately continuous in the main analysis; HC3 standard errors reduce sensitivity to heteroskedasticity in the relatively small sample. We also estimate robustness checks with a stricter sample definition, alternative income specifications, an alternative job satisfaction scale, ordered logit models using representative single items for each outcome, and IPW sensitivity models for complete-case selection. The ordered logit checks use L1A for job satisfaction, N1A for emotional exhaustion, N3A for personal accomplishment, and L2A for self-rated service quality, selected because each item directly reflects the corresponding outcome domain. All models are interpreted as associational rather than causal.

As a supplementary check, we estimated a seemingly unrelated regression (SUR) system for the four core outcomes using the same covariate structure as in the main OLS models. This step was designed to examine whether the focal association appears to vary across outcome equations, not to establish causal mechanisms. Because joint estimation requires a common complete-case sample, the SUR system was estimated on respondents with non-missing values for all four outcomes, the communication-and-cooperation index, the full control set, and city indicators (*N* = 236). Cross-equation Wald tests compare raw, unstandardized coefficients and are interpreted as exploratory, scale-dependent evidence rather than standardized effect-size comparisons.

## 4. Descriptive Statistics and Model Results

### 4.1. Sample Characteristics and Descriptive Statistics

The main analytical sample includes 262 medical social workers. Using the median of the perceived communication-and-cooperation index, we classify 191 respondents as the high communication-and-cooperation group and 71 as the low communication-and-cooperation group. The uneven split reflects clustering and ties at the upper end of the index rather than a balanced experimental grouping. The mean index is 4.087, suggesting generally favorable reported communication and cooperation and possible ceiling effects. The mean values for job satisfaction, emotional exhaustion, personal accomplishment, and self-rated service quality are 3.705, 1.246, 4.195, and 3.733, respectively. Descriptive statistics are reported in Table 1.

**Table 1:** Descriptive Statistics.

Variable	*N*	*Mean*	*SD*	Min	Max
Job satisfaction scale	261	3.705	0.629	1.0	5.0
Emotional exhaustion scale	261	1.246	1.032	0.0	6.0
Personal accomplishment scale	261	4.195	1.343	0.0	6.0
Self-rated service quality scale	260	3.733	0.516	2.444	5.0
Communication-and-cooperation index (physicians/nurses)	262	4.087	0.583	2.25	5.0
Communication with physicians	261	4.065	0.673	2.0	5.0
Cooperation from physicians	262	3.996	0.664	2.0	5.0
Communication with nurses	262	4.153	0.705	1.0	5.0
Cooperation from nurses	262	4.137	0.664	1.0	5.0
Organizational support	249	3.996	0.835	1.0	5.0
Autonomy/organizational fit	261	3.61	0.521	1.714	5.0
Professional identity	261	4.242	0.648	1.0	5.0
Female	262	0.74	0.439	0.0	1.0
Age	262	32.373	7.739	20.417	59.75
Education (ordinal)	259	3.988	0.795	1.0	5.0
Frontline service role	261	0.793	0.406	0.0	1.0
Management role	260	0.381	0.487	0.0	1.0
Supervisor role	259	0.216	0.412	0.0	1.0
Social work experience (years)	259	4.618	4.091	0.0	32.833
Monthly income, yuan (winsorized)	247	4565.889	2740.15	58.815	14,080.0

Note: Main analysis sample. Binary variables are coded 0/1. Ordinal and scale variables retain their survey ranges. Higher values on the four outcome scales indicate higher job satisfaction, emotional exhaustion, personal accomplishment, and self-rated service quality, respectively. Monthly income is reported in yuan in this descriptive table and rescaled per 1000 yuan in Table 2 regression models.

**Table 2:** Main Regression Results.

	Job Satisfaction	Emotional Exhaustion	Personal Accomplishment	Self-Rated Service Quality
Communication-and-cooperation index	0.260 ***	−0.186	0.416 **	0.151 **
	(0.078)	(0.119)	(0.194)	(0.068)
Female	−0.165	0.238	−0.280	−0.134
	(0.117)	(0.147)	(0.227)	(0.091)
Age	0.008	−0.004	0.031	0.013 *
	(0.007)	(0.013)	(0.019)	(0.007)
Education (ordinal)	0.019	0.075	0.135	0.128 **
	(0.073)	(0.115)	(0.134)	(0.054)
Frontline service role	0.229 **	−0.089	0.457 *	0.153 *
	(0.093)	(0.166)	(0.255)	(0.085)
Management role	−0.102	−0.041	−0.286	−0.016
	(0.104)	(0.156)	(0.253)	(0.092)
Supervisor role	0.004	0.190	0.320	0.199 **
	(0.132)	(0.218)	(0.243)	(0.095)
Social work experience (years)	0.009	−0.041 *	−0.027	−0.005
	(0.011)	(0.022)	(0.036)	(0.010)
Monthly income (winsorized, per 1000 yuan)	0.032	0.003	−0.010	0.011
	(0.019)	(0.034)	(0.046)	(0.016)
Open-ended contract	−0.089	−0.018	0.071	−0.110
	(0.101)	(0.196)	(0.277)	(0.097)
No written contract	0.179	−0.126	0.166	−0.004
	(0.117)	(0.174)	(0.269)	(0.109)
Dispatch employment	0.104	−0.339	0.970 ***	0.023
	(0.213)	(0.340)	(0.368)	(0.174)
Formal establishment	0.005	0.077	0.101	−0.065
	(0.105)	(0.164)	(0.238)	(0.089)
City fixed effects	Yes	Yes	Yes	Yes
Controls	Yes	Yes	Yes	Yes
Sample	Main	Main	Main	Main
*N*	237	237	237	236
*R* ^2^	0.253	0.280	0.233	0.197

Standard errors in parentheses. *** *p* < 0.01, ** *p* < 0.05, * *p* < 0.10.

**Figure 1:**
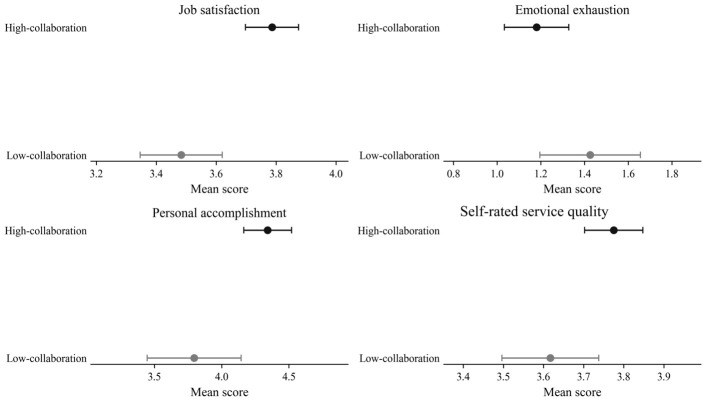
Descriptive group differences in key occupational outcomes. Note. Group membership is defined by the median split of the perceived communication-and-cooperation index. Points indicate group means, and error bars indicate 95% confidence intervals. The figure is descriptive only and does not adjust for covariates.

The two-group comparison is descriptive but consistent with the regression results that follow. The high communication-and-cooperation group reports higher mean job satisfaction, personal accomplishment, and self-rated service quality, and lower mean emotional exhaustion. These differences do not establish causation, but they show that the outcome distributions align with the main-model pattern. Figure 1 presents the mean differences with 95% confidence intervals.

The figure is descriptive and compares mean outcomes between high and low communication-and-cooperation groups with 95% confidence intervals; it does not represent a causal contrast.

### 4.2. Main Model Results

In the main models, after controlling for gender, age, education, role characteristics, years of practice, income, contract type, dispatch and establishment status, and city fixed effects, the perceived communication-and-cooperation index is positively associated with job satisfaction, personal accomplishment, and self-rated service quality. Its association with emotional exhaustion does not reach statistical significance in the main sample. Full estimates are reported in Table 2 (Main Regression Results).

All models include the full set of controls: sex, age, education, frontline service role, management role, supervisor role, social work experience, income, contract type, dispatch employment, and formal establishment status.

City fixed effects are included in all OLS models but omitted from the display.

HC3 heteroskedasticity-robust standard errors are used in all OLS specifications.

**Figure 2:**
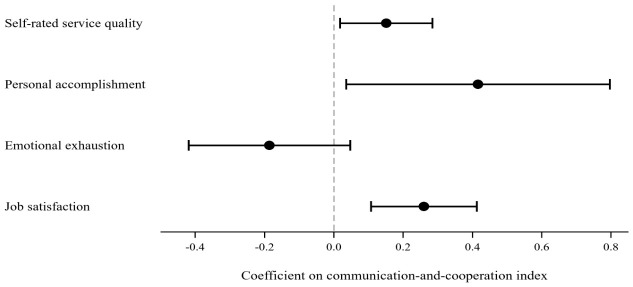
Main Regression Coefficients for the Communication-and-Cooperation Index.

The coefficient on the perceived communication-and-cooperation index is 0.2598 in the job satisfaction model, 0.4159 in the personal accomplishment model, and 0.1513 in the self-rated service quality model, with corresponding *p* values of 0.0010, 0.0335, and 0.0275. In the emotional exhaustion model, the coefficient is −0.1858 with *p* = 0.1207. These estimates indicate a fairly stable association between more positive communication and cooperation experiences and three favorable occupational outcomes, but a weaker and less stable relationship with emotional exhaustion. Figure 2 summarizes the unstandardized coefficient estimates and 95% confidence intervals.

The plot shows unstandardized OLS coefficients for the four-item communication-and-cooperation index with 95% confidence intervals based on HC3 robust standard errors; all models include the full control set and city fixed effects.

The pattern differs across outcomes. Job satisfaction, personal accomplishment, and self-rated service quality show stable positive associations with communication-and-cooperation experience. Emotional exhaustion shows a negative association in the expected direction, but the estimate is smaller and less stable.

We further examined this cross-outcome pattern through a supplementary SUR system estimated on the common complete-case sample (*N* = 236). The joint estimates preserved the main substantive pattern: the index remained positively associated with job satisfaction, personal accomplishment, and self-rated service quality, whereas its association with emotional exhaustion remained negative and comparatively weaker. On the raw outcome scales, the omnibus Wald test rejected equality of the index coefficients across the four equations (*Χ*^2^(3) = 13.46, *p* = 0.0037). Pairwise tests showed that the emotional-exhaustion coefficient differed from the coefficients for job satisfaction (*p* = 0.0005), personal accomplishment (*p* = 0.0038), and self-rated service quality (*p* = 0.0060). Differences among job satisfaction, personal accomplishment, and self-rated service quality were not statistically significant at conventional levels (*p* = 0.3363, *p* = 0.1410, and *p* = 0.0985, respectively). These tests compare unstandardized coefficients for outcomes measured on different scales and are therefore treated only as supplementary, scale-dependent evidence. We therefore use the SUR results to describe whether the cross-outcome pattern is consistent with the main models, rather than to rank the substantive size of the associations across outcomes.

### 4.3. Decomposed Model Results

To examine whether different components of the overall index behave differently, we decomposed the index into communication with physicians, cooperation from physicians, communication with nurses, and cooperation from nurses. The evidence is markedly weaker than in the overall-index models, and most component coefficients are not statistically significant. Only nurse cooperation shows a marginally significant positive association in the job satisfaction model (coefficient 0.2350, *p* = 0.0617), and only nurse communication shows a marginally significant positive association in the personal accomplishment model (coefficient 0.5259, *p* = 0.0532). These results do not identify stable component-specific effects. Full decomposed-model results are reported in Appendix A Table A2.

### 4.4. Moderation Results

We next examine whether work support, autonomy and organizational fit, and professional identity moderate the association between communication-and-cooperation experience and occupational outcomes. The moderation models do not show a stable pattern. Although these variables have significant main effects in some models, the corresponding interaction terms are not statistically significant. They therefore appear more plausibly as parallel work resources than as moderators of the focal association in this study. Full moderation results are reported in Appendix A Table A3.

### 4.5. Robustness Checks

Robustness checks use a stricter sample, alternative income specifications, the full job satisfaction scale, single-item ordered logit models, and IPW models for complete-case selection. The main OLS robustness results are summarized compactly in Table 3, with single-item ordered logit checks and IPW missingness sensitivity analysis reported in Appendix A Table A4 and Table A6.

**Figure A1:**
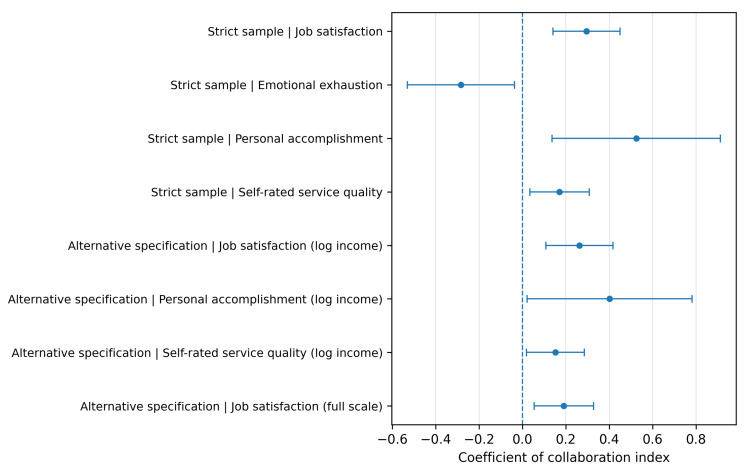
Robustness coefficients for the communication-and-cooperation index.

The robustness checks support the main pattern while also reinforcing caution about outcome-specific interpretation. For job satisfaction and self-rated service quality, the coefficients remain statistically significant in the stricter sample, under alternative income specifications, single-item ordered logit checks, and IPW adjustment for complete-case selection. Personal accomplishment remains significant in the stricter sample, the alternative income specification, and IPW models, although the representative single-item ordered logit check is positive but not statistically significant. Emotional exhaustion behaves differently. It is not significant in the main sample, under alternative income specifications, or in the IPW sensitivity models, but it becomes significantly negative in the stricter sample and marginally negative in the representative single-item ordered logit check. This result is consistent with an association in the expected direction, although it is less stable than the associations for the favorable evaluation outcomes. When the full job satisfaction scale replaces the five-item core measure, the communication-and-cooperation index remains significantly positive. Single-item ordered logit results are reported in Appendix A Table A4, the IPW missingness sensitivity analysis is reported in Appendix A Table A6, and Appendix A Figure A1 compares the direction, magnitude, and confidence intervals of the communication-and-cooperation index coefficient across robustness specifications.

## 5. Discussion

The most consistent results concern favorable occupational evaluations, especially job satisfaction and self-rated service quality, with personal accomplishment also supported in the main OLS, stricter-sample, alternative-income, and IPW specifications. The representative single-item check for personal accomplishment is positive but less precise, which is consistent with our broader caution that single items should be read as robustness checks rather than substitutes for the multi-item scale. Overall, the finding is best interpreted as evidence that smoother physician/nurse interfaces are linked to positive work appraisal and role enactment among medical social workers, not as proof that smoother communication and cooperation cause better occupational outcomes.

The supplementary cross-equation tests provide only scale-dependent support for this interpretation. Because the tests compare unstandardized coefficients across outcomes with different ranges, they should be read alongside the main OLS estimates and robustness checks rather than as stand-alone evidence of standardized effect-size differences. The safer conclusion is that the association appears more consistent for job satisfaction, personal accomplishment, and self-rated service quality than for emotional exhaustion.

This pattern is consistent with the nature of the outcomes. Job satisfaction, personal accomplishment, and self-rated service quality all concern how medical social workers judge the everyday operation of their work. Prior studies have shown that stronger teamwork, better communication, and smoother care coordination are associated with higher job satisfaction and more favorable self-rated quality evaluations among health professionals [19,20,27]. For medical social workers, smoother communication and stronger cooperation with physicians and nurses may reduce frictions in case advancement, allow professional judgments to enter team decisions, and facilitate the mobilization of service resources. These experiences are likely to appear first in workers’ assessments of their jobs, professional value, and self-rated service quality. Research on social work in integrated care offers a similar interpretation: collaborative environments are more likely to recognize and accept social workers’ professional roles, thereby strengthening professional visibility and value [6,10,39].

Emotional exhaustion follows a different pattern. The main coefficient is negative but not statistically significant, and significance appears only in the stricter sample. This suggests that better communication and cooperation may be linked to lower exhaustion, but the evidence is less stable than for the favorable evaluation outcomes. One reason is that exhaustion is a complex strain outcome shaped by workload, role conflict, support, organizational resources, and structural pressure [21,31,32,37,40,41,42,43,44,45]. This pattern suggests that exhaustion may be shaped less by the immediate physician/nurse interface alone than by the broader complexity of the care environment.

The care-complexity research programme initiated by Cesare, D’Agostino, and Cocchieri [46] offers a particularly valuable framework for interpreting this boundary. Their Nursing Reports study protocol argues that the complexity of care should be documented as a dimension distinct from medical complexity and examined through nursing minimum data capable of capturing nursing diagnoses, interventions, actions, and outcomes, such as length of stay. Although that programme is situated in paediatric nursing rather than medical social work, its conceptual implication is directly relevant here: multidisciplinary coordination is not merely a favourable workplace resource, but part of the infrastructure through which professionals translate multiple forms of patient need into organized care. Subsequent studies in this line further connect medical complexity with nursing complexity of care and link nursing complexity and health literacy to patient outcomes [47,48]. Our data cannot measure care complexity, medical complexity, health literacy, caseload, or patient-level coordination burden, so these findings should not be read as evidence about complexity mechanisms. They do, however, help explain why communication and cooperation with physicians and nurses is more consistently associated with positive work appraisals than with emotional exhaustion and suggest a future agenda linking patient-level complexity, multidisciplinary coordination, and worker outcomes.

The decomposed and moderation models should be read only as exploratory checks. Once the overall communication-and-cooperation experience is split into physician communication, physician cooperation, nurse communication, and nurse cooperation, most component coefficients are not statistically significant. The interaction terms for support, autonomy and organizational fit, and professional identity likewise do not identify a stable moderation pattern. These null and marginal findings do not reveal internal mechanisms; they mainly reinforce the decision to center the article on the overall four-item index and to treat supplementary models as boundary checks.

The practical implications are primarily organizational and should be stated cautiously. Hospitals that seek to support medical social workers’ job satisfaction, sense of accomplishment, and self-rated service quality could consider strengthening the interface between physicians, nurses, and medical social workers through formal referral protocols, routine case conferences that include social workers, shared discharge-planning procedures, written role descriptions, and feedback channels for reporting coordination barriers. These measures are plausible organizational supports, but the present cross-sectional study can show only associations; it cannot demonstrate that changing collaboration arrangements would improve outcomes. Chinese institutional accounts suggest that medical social work has developed unevenly across cities and hospital systems, which helps explain why the physician/nurse interface remains organizationally consequential in China [1,2].

Several limitations should be noted. The data are cross-sectional, and the medical subsample is based on convenience sampling; the analysis, therefore, addresses associations and should not be generalized directly to all medical social workers in China. The main sample depends on valid responses to the medical collaboration module, and complete-case estimation may introduce bias if missingness is related to income, employment conditions, organizational support, or institutional context. The added IPW sensitivity analysis addresses observed predictors of complete-case inclusion but cannot remove bias from unobserved determinants of missingness. Multiple imputation was considered but not used as the primary missing-data sensitivity analysis because the key missingness concerns involve a small medical-module sample, income validity, module routing, and sparse institutional/geographic cells. The study relies on self-reported survey measures, so common-method bias and self-report bias are possible. Some institutional and workload variables are unavailable in the public dataset, including hospital type, hospital level, department, caseload, administrative burden, supervision quality, and organizational resources. The emotional exhaustion result is sensitive to sample definition and should be revisited with larger samples or a more refined measurement. The supplementary decomposed, moderation, and SUR/Wald analyses are exploratory and should not be used to infer mechanisms. Longitudinal data, multi-source evidence, and richer organizational information would allow a more precise assessment.

## 6. Conclusions

Using the medical social work module of CSWLS2019, this study examined how medical social workers’ perceived communication and cooperation with physicians and nurses is associated with multiple occupational outcomes. The four-item index was positively associated with job satisfaction, personal accomplishment, and self-rated service quality, and these associations remained broadly stable across the stricter sample, alternative income specifications, and IPW adjustment for complete-case selection. Representative single-item ordered logit checks were strongest for job satisfaction and self-rated service quality, while the personal-accomplishment single-item check was positive but less precise. By contrast, the association with emotional exhaustion was negative in the main models but not statistically significant, reaching significance only in the stricter sample, and marginal significance in the single-item ordered logit check. Decomposed and moderation models provided limited evidence for component-specific effects or moderating mechanisms.

The article should be read as a practice-oriented exploratory study of associations. Its contribution is not a causal account of interprofessional collaboration as a multidimensional construct, but a focused comparison of how reported communication and cooperation with physicians and nurses relate to different occupational outcomes. The findings suggest that the physician/nurse interface is associated with whether medical social work can function as a recognized and sustainable part of team-based care in China.

## Figures and Tables

**Table 3:** Robustness Checks. Panel A reports strict-sample estimates; Panel B reports alternative specifications. Standard errors in parentheses.

**Panel A. Strict-Sample Estimates**				
	**Job Satisfaction**	**Emotional Exhaustion**	**Personal Accomplishment**	**Self-Rated Service Quality**
Communication-and-cooperation index	0.295 ***	−0.283 **	0.525 ***	0.171 **
	(0.079)	(0.126)	(0.198)	(0.070)
City fixed effects	Yes	Yes	Yes	Yes
Controls	Yes	Yes	Yes	Yes
Sample	Strict	Strict	Strict	Strict
*N*	226	226	226	225
R-squared	0.296	0.295	0.274	0.234
**Panel B. Alternative Specifications**				
	**Job satisfaction (log income)**	**Personal accomplishment (log income)**	**Self-rated service quality (log income)**	**Job satisfaction (full scale)**
Communication-and-cooperation index	0.263 ***	0.402 **	0.152 **	0.191 ***
	(0.079)	(0.194)	(0.068)	(0.070)
City fixed effects	Yes	Yes	Yes	Yes
Controls	Yes	Yes	Yes	Yes
Sample	Main	Main	Main	Main
*N*	238	238	237	237
R-squared	0.241	0.234	0.199	0.231

*** *p* < 0.01, ** *p* < 0.05. Only focal coefficients are shown; all models retain the full control set and city fixed effects unless otherwise noted. HC3 heteroskedasticity-robust standard errors are used in all OLS specifications.

## Data Availability

The CSWLS2019 data used in this study are restricted-access research data. They are not publicly available, but access may be requested through the CSWLS data management procedures and is subject to permission from the relevant data management committee.

## Appendix A

The appendix presents supplementary model results and robustness figures that are not discussed at length in the main text. The supplementary analyses referenced in the article correspond to Appendix A Table A1 (sample construction and missing-data diagnostic), Appendix A Table A2 (decomposed models), Appendix A Table A3 (compact moderation checks), Appendix A Table A4 (single-item ordered logit robustness checks), Appendix A Table A5 (supplementary raw-scale SUR joint estimation and cross-equation Wald tests), Appendix A Table A6 (IPW missingness sensitivity analysis for complete-case selection), and Appendix A Figure A1 (comparison of robustness coefficients).

**Table A1:** Sample Construction and Missing-Data Diagnostic.

Step	Communication-and-Cooperation Sample	*N* Retained	Use in Analysis
Main medical-module sample	Respondents with valid answers to Q2-Q5, the four physician/nurse communication and cooperation items	262	Main descriptive base and starting analytical sample
Stricter medical-condition sample	Main sample respondents who also satisfy at least one medical-condition indicator from routing or service-field information	249	Sensitivity analysis for sample definition
Main OLS complete cases	Non-missing outcome, communication-and-cooperation index, control variables, and city indicators	236–237	Model-specific listwise deletion in the four main outcome models
Moderation complete cases	Main model requirements plus the relevant moderator, such as support, autonomy/fit, or professional identity	229–237	Supplementary moderation models
SUR common complete cases	Non-missing values for all four outcomes, communication-and-cooperation index, controls, and city indicators	236	Appendix A Table A5 joint estimation and raw-scale Wald tests

**Table A2:** Decomposed Collaboration.

	Job Satisfaction	Emotional Exhaustion	Personal Accomplishment	Self-Rated Service Quality
Communication with physicians	0.014	−0.085	0.097	0.014
	(0.106)	(0.186)	(0.237)	(0.101)
Cooperation from physicians	0.099	−0.149	−0.043	0.078
	(0.105)	(0.167)	(0.226)	(0.096)
Communication with nurses	−0.074	−0.242	0.526 *	0.076
	(0.131)	(0.193)	(0.270)	(0.119)
Cooperation from nurses	0.235 *	0.278	−0.192	−0.024
	(0.125)	(0.220)	(0.262)	(0.106)
City fixed effects	Yes	Yes	Yes	Yes
Controls	Yes	Yes	Yes	Yes
Sample	Main	Main	Main	Main
*N*	236	236	236	235
*R* ^2^	0.273	0.296	0.239	0.196

* *p* < 0.10.

**Table A3:** Compact Moderation Checks.

**Panel A. Organizational Support Moderation**				
**Term**	**Job Satisfaction**	**Emotional Exhaustion**	**Personal Accomplishment**	**Self-Rated Service Quality**
Collaboration index (centered)	0.203 **	−0.173	0.284	0.104
	(0.078)	(0.129)	(0.189)	(0.070)
Organizational support (centered)	0.165 **	−0.116	0.279 **	0.133 ***
	(0.070)	(0.099)	(0.127)	(0.050)
Collaboration × organizational support	0.089	−0.074	0.186	−0.017
	(0.103)	(0.167)	(0.189)	(0.087)
City fixed effects	Yes	Yes	Yes	Yes
Controls	Yes	Yes	Yes	Yes
Sample	Main	Main	Main	Main
*N*	230	230	230	229
*R* ^2^	0.294	0.294	0.302	0.233
**Panel B. Autonomy/organizational fit moderation**				
**Term**	**Job satisfaction**	**Emotional exhaustion**	**Personal accomplishment**	**Self-rated service quality**
Collaboration index (centered)	0.180 **	−0.143	0.320	0.109
	(0.075)	(0.124)	(0.208)	(0.067)
Autonomy/organizational fit (centered)	0.562 ***	−0.301 **	0.675 **	0.313 ***
	(0.083)	(0.146)	(0.275)	(0.077)
Collaboration × autonomy/fit	−0.111	−0.052	−0.067	0.008
	(0.129)	(0.212)	(0.473)	(0.124)
City fixed effects	Yes	Yes	Yes	Yes
Controls	Yes	Yes	Yes	Yes
Sample	Main	Main	Main	Main
*N*	237	237	237	236
*R* ^2^	0.413	0.300	0.288	0.276
**Panel C. Professional identity moderation**				
**Term**	**Job satisfaction**	**Emotional exhaustion**	**Personal accomplishment**	**Self-rated service quality**
Collaboration index (centered)	0.211 ***	−0.174	0.352 *	0.112 *
	(0.071)	(0.119)	(0.209)	(0.064)
Professional identity (centered)	0.313 ***	−0.135	0.403 **	0.271 ***
	(0.071)	(0.111)	(0.170)	(0.057)
Collaboration × professional identity	0.080	0.090	0.122	0.011
	(0.115)	(0.195)	(0.388)	(0.101)
City fixed effects	Yes	Yes	Yes	Yes
Controls	Yes	Yes	Yes	Yes
Sample	Main	Main	Main	Main
*N*	237	237	237	236
*R* ^2^	0.335	0.288	0.265	0.293

Note: Coefficients are unstandardized OLS interaction terms from models with centered communication-and-cooperation index and centered moderator. HC3 heteroskedasticity-robust standard errors are shown in parentheses. All models include the full covariate set and city fixed effects. *** *p* < 0.01, ** *p* < 0.05, * *p* < 0.10.

**Table A4:** Ordered Logit Robustness Checks.

	Job Satisfaction Item (L1A)	Emotional Exhaustion Item (N1A)	Personal Accomplishment Item (N3A)	Self-Rated Service Quality Item (L2A)
Communication-and-cooperation index	0.682 ***	−0.447 *	0.365	0.601 **
	(0.253)	(0.242)	(0.238)	(0.273)
City indicators	Yes	Yes	Yes	Yes
Controls	Yes	Yes	Yes	Yes
Sample	Main	Main	Main	Main
*N*	237	237	237	236

Standard errors in parentheses. *** *p* < 0.01, ** *p* < 0.05, * *p* < 0.10. Only the focal coefficient is shown; all models include the same controls and city indicators as the main specification. The ordered logit checks use representative single items: L1A, N1A, N3A, and L2A.

**Table A5:** Supplementary SUR Joint Estimation and Cross-Equation Wald Tests.

**Panel A. SUR Estimates for the Communication-and-Cooperation Index**					
**Outcome**	***N***	**Coef.**	**Robust SE**	**95% CI**	***p***
Job satisfaction	236	0.2553	0.0673	[0.1234, 0.3873]	0.0001
Emotional exhaustion	236	−0.1928	0.1055	[−0.3996, 0.0140]	0.0677
Personal accomplishment	236	0.4113	0.1681	[0.0819, 0.7408]	0.0144
Self-rated service quality	236	0.1513	0.0598	[0.0342, 0.2685]	0.0113
**Panel B. Cross-equation Wald tests for equality of raw-scale communication-and-cooperation-index coefficients**					
**Restriction**	**df**	**Difference**	**SE diff.**	**Chi-square**	***p***
All four coefficients equal	3	--	--	13.4625	0.0037
Job satisfaction = emotional exhaustion	1	0.4481	0.1283	12.2011	0.0005
Job satisfaction = personal accomplishment	1	−0.1560	0.1623	0.9243	0.3363
Job satisfaction = self-rated service quality	1	0.1040	0.0706	2.1672	0.1410
Emotional exhaustion = personal accomplishment	1	−0.6041	0.2089	8.3598	0.0038
Emotional exhaustion = self-rated service quality	1	−0.3441	0.1252	7.5569	0.0060
Personal accomplishment = self-rated service quality	1	0.2600	0.1573	2.7303	0.0985

Note: SUR system estimated on the common complete-case sample (*N* = 236) with the same covariates and city indicators as the main specification.

Panel A reports robust standard errors and 95% confidence intervals for the collaboration-index coefficient in each outcome equation. Panel B reports cross-equation Wald tests of equality for the raw, unstandardized collaboration-index coefficient; the Difference column is calculated as the first-listed outcome coefficient minus the second-listed outcome coefficient. Because the outcomes have different native measurement scales, these tests are exploratory and should not be read as standardized effect-size comparisons.

**Table A6:** Inverse-probability weighted sensitivity analysis for complete-case selection.

Outcome	Main Complete-Case OLS	IPW OLS	*N*	Stabilized Weight Mean	Stabilized Weight Range
Job satisfaction	0.260 *** (0.078)	0.253 *** (0.077)	237	0.991	0.923–1.831
Emotional exhaustion	−0.186 (0.119)	−0.161 (0.126)	237	0.991	0.923–1.831
Personal accomplishment	0.416 ** (0.194)	0.400 ** (0.191)	237	0.991	0.923–1.831
Self-rated service quality	0.151 ** (0.068)	0.149 ** (0.066)	236	0.991	0.919–2.086

Notes: Entries are coefficients for the four-item physician/nurse communication-and-cooperation index, with HC3 robust standard errors in parentheses. All models include the same individual controls and city fixed effects as the main OLS models. Stabilized inverse-probability weights were estimated from a ridge-logistic model of inclusion in each outcome-specific complete-case analytic sample, using variables observed for the full main sample: collaboration index, gender, age, Q2-Q5 complete-response status, questionnaire routing indicators, number of valid collaboration items, and city indicators. ** *p* < 0.05, *** *p* < 0.01.

The following appendix figure summarizes the direction, magnitude, and confidence intervals of the communication-and-cooperation index coefficient across robustness specifications.
